# tRNA-Derived Fragment tRF-Glu-TTC-027 Regulates the Progression of Gastric Carcinoma *via* MAPK Signaling Pathway

**DOI:** 10.3389/fonc.2021.733763

**Published:** 2021-08-23

**Authors:** Weiguo Xu, Bin Zhou, Juan Wang, Li Tang, Qing Hu, Jian Wang, Huanhuan Chen, Junyu Zheng, Feng Yan, Huanqiu Chen

**Affiliations:** ^1^Department of General Surgery, Jiangsu Cancer Hospital & The Affiliated Cancer Hospital of Nanjing Medical University & Jiangsu Institute of Cancer Research, Nanjing, China; ^2^Department of Oncology, Jiangsu Cancer Hospital & The Affiliated Cancer Hospital of Nanjing Medical University & Jiangsu Institute of Cancer Research, Nanjing, China; ^3^Department of Clinical Laboratory, Jiangsu Cancer Hospital & The Affiliated Cancer Hospital of Nanjing Medical University & Jiangsu Institute of Cancer Research, Nanjing, China

**Keywords:** tRF-Glu-TTC-027, MAPK signaling pathway, gastric cancer (GC), tRNA-derived small RNA (tsRNA), tRNA-derived RNA fragments (tRFs)

## Abstract

Transfer RNA-derived RNA fragments (tRFs) belong to non-coding RNAs (ncRNAs) discovered in most carcinomas. Although some articles have demonstrated the characteristics of tRFs in gastric carcinoma (GC), the underlying mechanisms still need to be elucidated. Meanwhile, it was reported that the MAPK pathway was momentous in GC progression. Thus we focused on investigating whether tRF-Glu-TTC-027 could act as a key role in the progression of GC with the regulation of the MAPK pathway. We collected the data of the tRNA-derived fragments expression profile from six paired clinical GC tissues and corresponding adjacent normal samples in this study. Then we screened tRF-Glu-TTC-027 for analysis by using RT-PCR. We transfected GC cell lines with tRF-Glu-TTC-027 mimics or mimics control. Then the proliferation, migration, and invasion assays were performed to assess the influence of tRF-Glu-TTC-027 on GC cell lines. Fluorescence *in situ* hybridization assay was conducted to confirm the cell distribution of tRF-Glu-TTC-027. We confirmed the mechanism that tRF-Glu-TTC-027 influenced the MAPK signaling pathway and observed a strong downregulation of tRF-Glu-TTC-027 in clinical GC samples. Overexpression of tRF-Glu-TTC-027 suppressed the malignant activities of GC *in vitro* and *in vivo*. MAPK signaling pathway was confirmed to be a target pathway of tRF-Glu-TTC-027 in GC by western blot. This is the first study to show that tRF-Glu-TTC-027 was a new tumor-suppressor and could be a potential object for molecular targeted therapy in GC.

## Introduction

According to the report issued by the National Cancer Registry in 2015, the statistical results of the disease show that the number of cases of gastric cancer (GC) in China is about 679,100, second only to lung cancer (733,300) and higher than esophageal cancer (477,900) and liver cancer (466,100). The total number of deaths due to gastric cancer is approximately 498,000 which is also second only to lung cancer, ranking second in tumor mortality. Despite the global downward trend of incidence and mortality of gastric cancer, the corresponding data is still at a relatively high level in China. Therefore, for a long time in the future, gastric cancer will still be one of the major public health issues in China (1, 2).

tRNA-derived small RNAs (tsRNAs) can be classified into two kinds of ncRNAs, tRFs and tiRNAs. Transfer RNA-derived RNA fragments (tRFs) are derived from mature or precursor tRNAs and are composed of 14–30 nt in length approximately. tiRNAs, consist of 29–50 nt in length, are generated by spliced anticodon loop of tRNA (3). Fu et al. reported that the generation of tiRNAs has been discovered to occur through specific ribonucleases such as Angiogenin (4, 5). Transfer RNA-derived RNA fragments (tRFs) and transfer RNA-derived stress-induced RNA (tiRNAs) belong to ncRNAs. These RNAs can be both produced under the circumstance of stress (6). It should be pointed out that tRFs and tiRNAs are reported to emerge from ribonucleolytic splicing of tRNAs by Dicer (7) and RNase Z (8). Meanwhile, tiRNAs can be classified into 5’ tiRNAs and 3’ tiRNAs. tRNA derivatives are found related to Argonaute proteins, and they could target mRNA 3’UTR to act as the RNA silencer (9, 10). Some studies have revealed that tRNA derivatives could influence tumor proliferation, DNA damage repair, and the regulation of tumor-related signaling pathways *via* mRNA silencing (6, 11–13). Generally, it showed that tRFs and tiRNAs could act as novel potential biomarkers and cancer treatment targets.

Concerning this study, we screened out several differential expressed tRNA derivatives based on high-throughput sequencing. Through the exploration of the biological functions of GC cell lines and the verification of the expression level of gastric cancer tissues, we selected tRF-Glu-TTC-027 as the target of our further research. Little is known about the function of tRF-Glu-TTC-027 in gastric cancer as it is a brand new tRNA derivative. With the multi-dimensional bioinformatics analysis of the target genes of tRNA derivatives in the high-throughput sequencing files, we discovered a few signaling pathways responsible for the change of the biological characteristics, especially the MAPK signaling pathway. In our study, tRF-Glu-TTC-027 was downregulated in gastric cancer tissues, with an inverse correlation with tumor size and histology. Meanwhile, tRF-Glu-TTC-027 declined the progression of GC cells *in vitro* and suppressed tumor growth of gastric cancer *in vivo*. Our study concludes that tRF-Glu-TTC-027 plays an inhibitory role in gastric cancer.

## Materials and Methods

### Tissue Specimens

All the collected human gastric cancer tissues came from The Affiliated Cancer Hospital of Nanjing Medical University between 2016 and 2017, and the patients’ informed consents were obtained. Our hospital’s ethics committee evaluated and authorized this experiment prior to its implementation (NYDLS-2019-919). At the same time, we carefully read and abided by the Declaration of Helsinki. After the gastric cancer tissues were obtained, we strictly abided by the relevant tissue handling specifications and froze them in the refrigerator at -80°C.

### tRFs&tiRNAs Expression Analysis

In our high-throughput sequencing files, the expression data of tRFs and tiRNAs were presented in counts and normalized to total aligned reads (CPM). The R package edgeR was utilized to screen the tRFs and tiRNAs. We used Fold Change (cutoff 1.5) and P-value (cutoff 0.05) for screening differentially expressed tRFs and tiRNAs. Principal Component Analysis (PCA), Correlation Analysis, Venn plots, Hierarchical clustering, Scatter plots, and Volcano plots were analyzed in R software or Perl environment. We utilized the GO and KEGG analysis on the potential targets of tRNA derivatives. Analysis of Biological Process (BP), Cellular Component (CC), and Molecular Function (MF) was presented with R software in GO analysis. P < 0.05 was considered significantly enriched in a certain process. Pathway clustering was conducted using KEGG analysis. We applied the enrichment score to indicate the significance of correlation. To elucidate the GC-related pathways more comprehensively, we acquired microarray profile GSE65801 in the GEO database and TCGA-STAD database. We then undertook the KEGG and GSEA analysis to further investigate the possible biological characteristics of GC.

### Cell Culture and Transfection

We obtained the GC cell lines NCI-N87, HGC-27, BGC-823, SGC-7901, and GES-1 from the Type Culture Collection of the Chinese Academy of Sciences (Shanghai, China). GES-1 cell line was grown in 10% FBS DMEM medium. Medium for HGC-27, BGC-823, and SGC-7901 was RPMI 1640 with 10% FBS, for NCI-N87 was RPMI 1640 medium with 15% FBS. All media were purchased from ThermoFisher. We incubated the cell lines in a humidified incubator at 37°C with 5% CO2. The GC cells were seeded in a 6-well plate at a cell density of 20%-40%. 24 hours later, we used Lipofectamine 2000 (ThermoFisher, USA) to transfect tRF mimics or inhibitors (Ribobio, Guangzhou, China) (50 nM) into GC cells. We selected p38 MAPK pathway inhibitor (p38 MAPK-IN, MCE, USA) to act as the positive control of tRF-Glu-TTC-027. The detailed steps are available in our previous study (14). The sequences of the tRF mimic and corresponding control group were listed in **Table 1**.

**Table 1:** The sequence of primers and tRF mimics.

Names	Sequence (5’-3’)
tRF-Glu-TTC-027 mimics	UGACUGGACCUUUCUUU
scramble mimics control	ACUUGGUCCGUUCUUAU
ELK4-F	TCCAAAGATGTGGAGAATGGAG
ELK4-R	GAGTGTATGTAGTCATTGCGGCT
TGFB2-F	AGTCATACCACCTTTCCGATTG
TGFB2-R	ACGGCACAGGGATTTCTTCTA
U6-F	CTCGCTTCGGCAGCACA
U6-R	AACGCTTCACGAATTTGCGT
GAPDH-F	ACCCACTCCTCCACCTTTGAC
GAPDH-R	TGTTGCTGTAGCCAAATTCGTT

F, forward; R, reverse. The sequence information of all siRNA controls, ASO controls, miRNA primers, and probes is not public according to the declaration of RiboBio (Guangzhou, China).

### RNA Extraction, Reverse Transcription, and Quantitative Real-Time PCR

We put the preserved GC tissue into the TRIzol solution (Life Technologies, USA) for RNA extraction. After the process of chloroform, isopropanol, ethanol, we got the relatively pure RNA. During the process, the RNA solution was centrifuged several times. Then we applied nanodrop to determine the RNA concentration and the OD values of 260/280 absorbance ratios were between 1.8 and 2.0. Next, we performed RNA reverse transcription (RT) using the riboSCRIPT Reverse Transcription Kit (RiboBio, Guangzhou, China). Reaction conditions for RT were 60 min at 42°C and 10 min at 70°C. Finally, we applied LightCycler 1.5 (Roche, Switzerland) to manipulate PCR in a 20 μL reaction system (2 μL cDNA, 6.4 μL DEPC, 10 μL SYBR Green Mix, 0.8 μL forward primer, and 0.8 μL reverse primer). PCR reaction conditions were 95°C for 30 sec, 40 cycles of 95°C for 5 sec and 60°C for 20 sec. U6 was used as an internal control. The detailed steps are available in our previous study (14). The primers used in qPCR were presented in **Table 1**. The relative expression was analyzed with the 2^-ΔΔCt^ method.

### Fluorescent *In Situ* Hybridization (FISH)

After the cells were treated with 4% paraformaldehyde, the cell membranes were permeabilized by Triton-100 (Beyotime, Shanghai, China) for 5 min at 4°C. Next, we added the hybridization solution mixed with Cy3-labelled tRF-Glu-TTC-027 to the permeabilized cells to locate the tRF-Glu-TTC-027 in GC cells. We stained the GC nuclei with DAPI and captured the pictures with a fluorescence microscope.

### Cell Proliferation Assay and Colony Formation Assay

A certain number (2 x 10^3^) of transfected GC cells were transferred to a 96-well plate, and then we added 10ul CCK8 solution (Dojindo, Japan) into each well to measure the OD value in 450 nm by the microplate reader after 2 hours of incubation at 0, 24, 48, 72 and 96 hours. We added 1,000 transfected cells into each well of the 6-well plate, and these GC cells were cultured for 10-14 days. Subsequently, we terminated the cultivation with 4% paraformaldehyde as soon as the macroscopic clones were observed. The 1% crystal violet was used to stain the clones and the numbers were counted using Image J software.

### Edu Assay

Cell-Light EdU Apollo *In Vitro* Kit (Ribobio, Guangzhou, China) for EdU assay was utilized to compare the growth ability of the transfected GC cell. NCI-N87 and HGC-27 cell lines were transfected with tRF-Glu-TTC-027 mimics or mimics control, then 4 x 10^3^ transfected cells were transferred into each well of the 96-well plate. We diluted the EdU solution with complete medium to prepare enough 50 μM EdU medium. And EdU medium was then added into each well to incubate the cells for two hours. After the fixation, permeabilization, and staining, we captured the images with a fluorescence microscope.

### Migration and Invasion Assays

For wound healing assay, GC cells were seeded in a 6-well plate with 1.5×10^6^ cells/well 24 hours before the experiment, and the tRF-Glu-TTC-027 mimics group and mimics control group were plated in three wells each. On the day of the experiment, we used a pipette tip to make a “+” mark in the 6-well plate and washed the exfoliated cells with PBS buffer. Then we took a picture and recorded the width of the scratch, and added 2 mL of complete medium to each well for culture. After 24h, we captured a picture and recorded the width of the scratch again. Percentage of gap closure = (0h scratch width - 24h scratch width)/0h scratch width x 100%. For transwell migration and invasion assays, we took GC cells in the logarithmic growth phase to prepare the cell suspension with a cell density of 2 x 10^6^/mL using the serum-free medium. The upper layer of the transwell chamber with or without matrigel was then filled with 200 μL cell suspension, and the lower chamber was filled with 600 μL complete medium. After the incubation for 24 h in the incubator, we removed the liquid in the chamber’s upper layer and carefully cleaned off the non-penetrated cells with a cotton swab. The polycarbonate membrane of the chamber was stained with hematoxylin, and we counted the number of penetrated cells under an optical microscope. Our previous research has described the specific steps in detail (14).

### Cell Cycle Determination

NCI-N87 and HGC-27 cells were first harvested after 48 hours of transfection and the cell suspension was then centrifuged. Subsequently, we washed the cell pellet three times with ice-cold PBS and conducted the fixation with 70% ethanol. Afterward, we resuspended the cells in the PI-staining solution to perform the flow cytometry analysis.

### Western Blot

The supernatant of the cell lysates was transferred to another clean centrifuge tube, and we used the BCA protein assay kit (ThermoFisher, USA) to detect the protein concentration. Then we performed gel electrophoresis (10% SDS-PAGE) and transferred the protein to a polyvinylidene fluoride membrane. The membrane was immersed in 5% skimmed milk for one hour and treated with the primary antibodies at 4°C with gentle tremors overnight. Next, the secondary antibody was added to block the membrane for two hours. We used the chemiluminescence method to develop and measure the gray band. Antibody dilutions and manufacturers are provided in **Table 2**.

**Table 2:** Information of antibodies for Western blot, immunofluorescence staining, and IHC.

Antibody	Manufacturer	Item NO.	Species	Dilution (primary)
ERK1/2	Servicebio	GB11560	Rabbit	1: 1000
p-ERK1/2	CST	4370	Rabbit	1: 1000
JNK	proteintech	24164-1-AP	Rabbit	1: 1000
p-JNK	CST	4668	Rabbit	1: 1000
p38	abcam	ab32142	Rabbit	1: 1000
p-p38	CST	4511	Rabbit	1: 1000
c-Myc	proteintech	10828-1-AP	Rabbit	1: 1000
CDK2	BOSTER	BM0463	Mouse	1: 1000
CyclinD1	proteintech	60186-1-LG	Mouse	1: 1000
Elk1	proteintech	27420-1-AP	Rabbit	1: 1000
p-Elk1	Affinity	AF3212	Rabbit	1: 1000
ELK4	proteintech	14666-1-AP	Rabbit	1: 1000
TGFB2	proteintech	19999-1-AP	Rabbit	1: 1000
Ki67	abcam	ab16667	Rabbit	1: 200
β-actin	Servicebio	GB12001	Mouse	1: 1000

### Subcutaneous Xenograft Experiments

We obtained the five-week-old Balb/c female nude mice from the Shanghai Experimental Animal Center of the Chinese Academic of Sciences (Shanghai, China). The mice were randomly assigned to NS, tRF-Glu-TTC-027 NC, tRF-Glu-TTC-027 agomir groups (tRF-Glu-TTC-027 overexpression), and 1.2 x 10^6^ treated NCI-N87 cells were then subcutaneously injected into their right flanks. tRF-Glu-TTC-027 NC and tRF-Glu-TTC-027 agomir were designed by Ribobio company (Ribobio, Guangzhou, China). We measured the tumor sizes every three days. 40 days later, mice were sacrificed and the tumors were resected and sliced for IHC analysis and immunofluorescence assay. The tumor weight was measured and the volume was determined using the formula: 1/2 x (length x width^2^).

### Immunohistochemistry (IHC)

The paraffin-embedded tissue sections were deparaffinized and dehydrated. Then the sections were blocked with immunostaining blocking solution for 15 minutes at RT. Afterward, they were incubated in the Ki67 primary antibody (**Table 2**) and secondary antibody for one hour, respectively. DAB substrate was used for staining. We counter-stained the cell nucleus with hematoxylin for two minutes and dehydrated the sections with ethanol. Images were captured by a microscope.

### Immunofluorescence Staining

The tissue sections were infiltrated with PBS and rewarmed for 20 minutes. Then permeabilization was conducted using 0.5% Triton X-100 (prepared in PBS) at RT for 20 minutes. Next, we applied goat serum to block the tissue section for 30 minutes, and then the diluted primary antibody (Ki67) and fluorescent secondary antibody were used to incubate with each slide successively. Afterward, we immersed the slides with PBST and stained the specimens with DAPI. All the collected images were observed under a fluorescence microscope.

### Statistical Analysis

We applied SPSS 19.0 (IBM, Chicago, USA) and GraphPad Prism 6 (San Diego, CA, USA) to deal with the data. Data were presented as mean ± SD. The Chi-square test or Fisher’s exact test was applied to compare the composition ratio of two or more samples and analyze the correlation of two categorical variables. The Kaplan-Meier technique was used to evaluate the follow-up data, and the log-rank test was performed to assess the differences between groups. To compare the means of numerical variables, we conducted the Student’s t-test or one-way ANOVA test. P < 0.05 was regarded as statistically significant.

## Results

### Expression Profiles of tRNA-Derived Fragments

**Figure 1:**
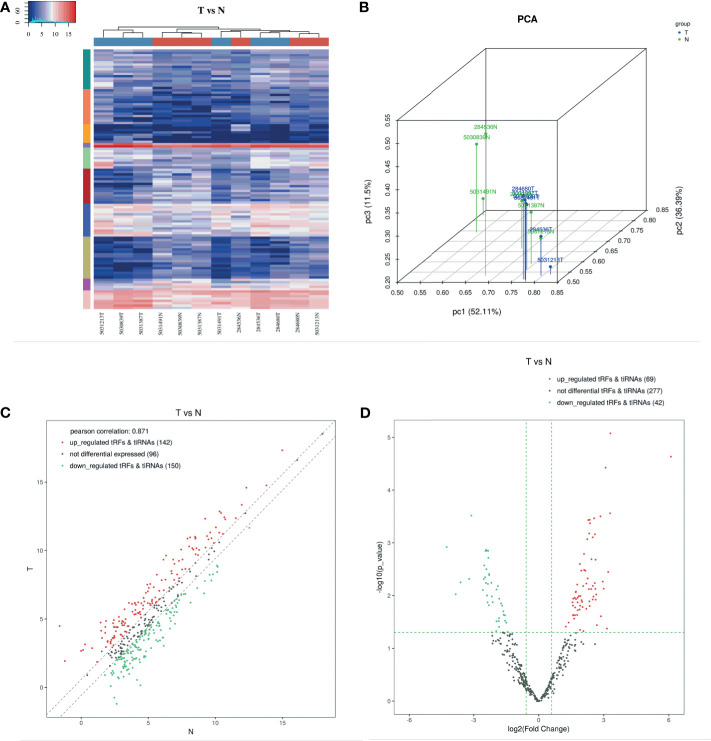
Expression Profiles of tRNA-derived Fragments. **(A)** The hierarchical clustering heatmap for tRF & tiRNA. In this heatmap, the upper color bar represents the group classification of gastric cancer tissues and NATs. The left color bar of the panel represents the cluster analysis of differentially expressed tsRNAs based on K-means. Generally, red indicates the high expression level, and blue indicates the low expression level. **(B)** Primary component analysis. The result of PCA analysis shows a distinguishable tRF & tiRNA expression profiling among the GC and NATs samples. The figure is an overview of samples correlations. **(C)** The scatter plot between two groups for tRF & tiRNA. 142 up-regulated tRFs & tiRNAs, 150 down-regulated tRFs & tiRNAs, and 96 not differential expressed tRFs & tiRNAs were screened out. The Pearson correlation between GC and NATs was 0.871. **(D)** The volcano plot of tRF & tiRNA. The volcano plot is based on CPM values of tsRNAs, and we found that 69 up-regulated tRFs & tiRNAs, 42 down-regulated tRFs & tiRNAs, and 277 not differential tRFs & tiRNAs were uncovered.

Differentially expressed tsRNAs were provided in **Supplementary Tables 1** and **2**. We performed hierarchical clustering for tsRNAs differential expression data in high-throughput sequencing files. The results indicated that tsRNAs were significantly differentially expressed between gastric cancer and non-tumor adjacent tissue specimens (NATs) (**Figure 1A**). The result of PCA analysis shows a distinguishable tRF & tiRNA expression profiling among the GC and NATs samples. The figure is an overview of samples correlations using the R scatterplot3d package (**Figure 1B**). In terms of the results of the scatter plot, 142 up-regulated tRFs & tiRNAs, 150 down-regulated tRFs & tiRNAs, and 96 not differential expressed tRFs & tiRNAs were screened out. The Pearson correlation between GC and NATs was 0.871 (**Figure 1C**). The volcano plot is based on CPM values of tsRNAs, and we found that 69 up-regulated tRFs & tiRNAs, 42 down-regulated tRFs & tiRNAs, and 277 not differential tRFs & tiRNAs were uncovered, which was taken into analysis with the criteria of the p-value (<0.05) (**Figure 1D**).

### GO and KEGG Analysis

**Figure 2:**
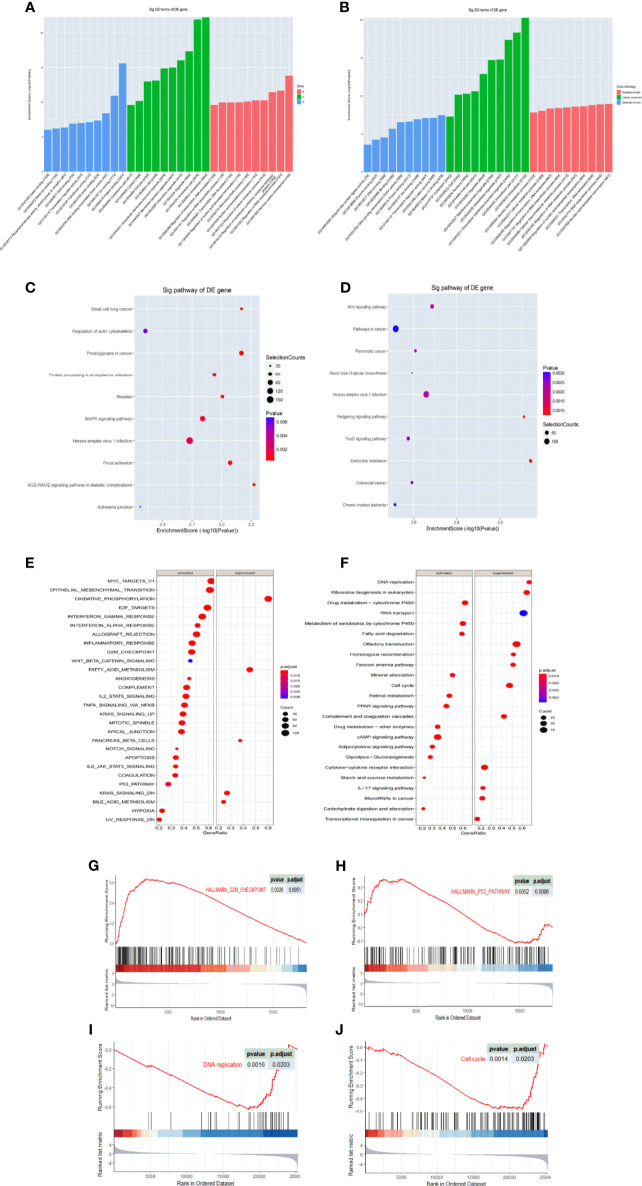
GO and KEGG enrichment analysis of tRF-Glu-TTC-027 target genes. **(A)** Column chart of up-regulated target genes in GO analysis; **(B)** Column chart of down-regulated target genes in GO analysis. The enrichment score was denoted on Y-axis, and the MF, CC, and BP terms were showed on X-axis. **(C)** KEGG pathway analysis showed 10 significant pathways of up-regulated target genes in gastric cancer tissues. **(D)** KEGG pathway analysis showed 10 significant pathways of down-regulated target genes in gastric cancer tissues. **(E, F)** KEGG pathway analysis of GC in TCGA and GEO databases. **(G–J)** GSEA analysis of GC in TCGA and GEO databases. DEGs, differentially expressed genes.

We then predicted the target gene of tRF-Val-CAC-016, tRF-Glu-TTC-027, tRF-Glu-TTC-026, tRF-Ser-TGA-011, tiRNA-Pro-TGG-001, tRF-Ser-GCT-113, tiRNA-Val-CAC-001, tiRNA-His-GTG-001, tRF-Glu-TTC-017, and tiRNA-Asp-GTC-001 through GO and KEGG pathways. We conducted GO and KEGG analysis separately according to the expression level of the tsRNAs. Ten most statistically significant targets of GO and KEGG analysis of the down-regulated group (tRF-Val-CAC-016, tRF-Glu-TTC-027, tRF-Glu-TTC-026, tRF-Ser-TGA-011, tiRNA-Pro-TGG-001, tRF-Ser-GCT-113) are presented in **Figures 2A, C**, and the results of the up-regulated group (tiRNA-Val-CAC-001, tiRNA-His-GTG-001, tRF-Glu-TTC-017, tiRNA-Asp-GTC-001) are presented in **Figures 2B, D**. The most enriched biological process (BP) was nervous system development (GO: 0007399) in the down-regulated group and nucleic acid-templated transcription (GO: 0097659) in the up-regulated group. Concerning the CC analysis, it indicated that enrichment mainly occurred at intracellular (GO:0005622) in both groups. For MF, the analysis presented that the enriched function was protein binding (GO: 0005515) in the down-regulated group, and metal ion binding (GO: 0046872) in the up-regulated group (**Figures 2A, B** **)**. In the KEGG pathways analysis, AGE-RAGE signaling pathway in diabetic complications (hsa04933), Small cell lung cancer (hsa05222), Proteoglycans in cancer (hsa05205), Focal adhesion (hsa04510), Measles (hsa05162), Protein processing in the endoplasmic reticulum (hsa04141), MAPK signaling pathway (hsa04010), Herpes simplex virus 1 infection (hsa05168), Regulation of actin cytoskeleton (hsa04810), and Adherens junction (hsa04520) were significantly enriched in the down-regulated group (**Figure 2C**). Endocrine resistance (hsa01522), Hedgehog signaling pathway (hsa04340), Wnt signaling pathway (hsa04310), Herpes simplex virus 1 infection (hsa05168), Pancreatic cancer (hsa05212), Mucin type O-glycan biosynthesis (hsa00512), Colorectal cancer (hsa05210), FoxO signaling pathway (hsa04068), Pathways in cancer (hsa05200), and Chronic myeloid leukemia (hsa05220) were meaningfully enriched in the up-regulated group (**Figure 2D**). We then searched TCGA and GEO databases to compare the bioinformatic analysis with present sequencing data, and we discovered that proliferation-related pathways were significantly enriched in KEGG analysis (**Figures 2E, F** **)**. Meanwhile, the G2M checkpoint and p53 pathway in the GEO database (**Figures 2G, H** **)**, DNA replication and cell cycle pathway in the TCGA database (**Figures 2I, J** **)** were significantly enriched in GSEA analysis. Above all, the MAPK signaling pathway was directly selected according to the bioinformatic analysis and the following biological assays of tRF-Glu-TTC-027.

### The Expression of tRF-Glu-TTC-027 Was Down-Regulated in GC Significantly

**Figure 3:**
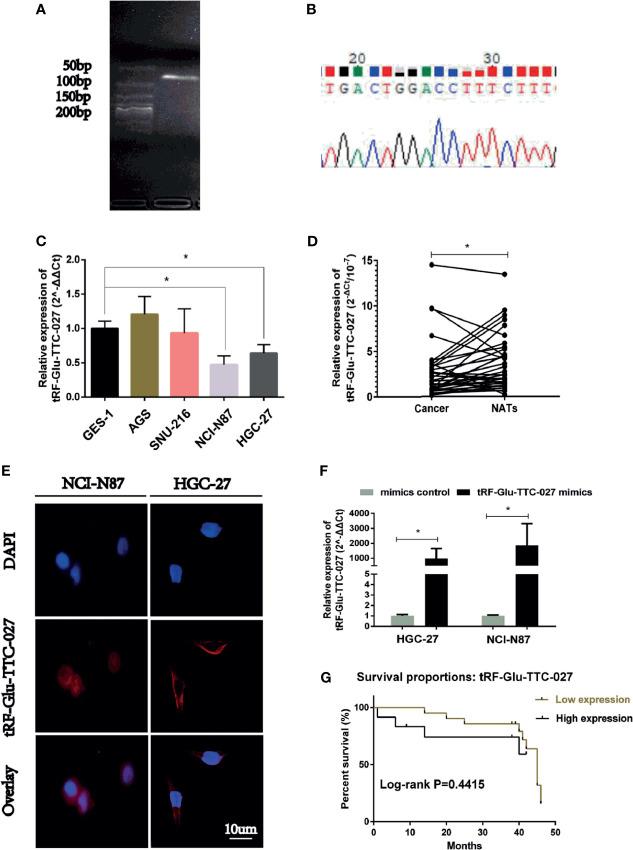
The expression of tRF-Glu-TTC-027 was significantly down-regulated in GC tissues and cell lines. **(A)** The primers of tRF-Glu-TTC-027 were designed, and gel electrophoresis was used to further clarify the PCR product for tRF-Glu-TTC-027. **(B)** The result of Sanger sequencing of tRF-Glu-TTC-027 PCR product. **(C)** The inhibitory effect of tRF-Glu-TTC-027 on GC cells was detected by PCR. NCI-N87 and HGC-27 were picked out as the low expression of tRF-Glu-TTC-027. **(D)** Through PCR detection of 35 pairs of gastric cancer tissues, we found that tRF-Glu-TTC-027 was less expressed in tumor specimens than NATs. **(E)** FISH detection of tRF-Glu-TTC-027 in NCI-N87 and HGC-27 cells. The cy3 labeled tRF-Glu-TTC-027 was red, while the nucleus stained by DAPI was blue. **(F)** Designed tRF-Glu-TTC-027 mimics were capable of efficiently up-regulating the expression of tRF-Glu-TTC-027 in NCI-N87 and HGC-27. **(G)** The prognosis of tRF-Glu-TTC-027 in GC patients, P =0.4415. (Data are presented as mean ± SD. *P < 0.05, Student’s t-test, Scale bar = 10 µm).

We used the gel electrophoresis to further clarify the PCR product for tRF-Glu-TTC-027 (**Figure 3A**). The results of Sanger sequencing confirmed the validity of the primers and the authenticity of the PCR product sequence of the tRF-Glu-TTC-027 (**Figure 3B**). Next, we evaluated 35 pairs of tissues with RT-PCR, and tRF-Glu-TTC-027 was tested a low expression of great significance in GC compared with NATs, and so is in the detection of GC cells (NCI-N87, HGC-27) compared with GES-1 (**Figures 3C, D** **)**. FISH assay confirmed that tRF-Glu-TTC-027 was most concentrated in the cytoplasm (**Figure 3E**). Meanwhile, designed tRF-Glu-TTC-027 mimics were capable of efficiently up-regulating the expression of tRF-Glu-TTC-027 (**Figure 3F**). Subsequently, we uncovered that the expression level of tRF-Glu-TTC-027 was significantly associated with tumor size and histological grade in terms of clinicopathological studies (**Table 3**). Through the analysis of the PCR data of 35 pairs of GC tissues and the follow-up data of corresponding patients, we found that tRF-Glu-TTC-027 was not significantly related to the prognosis of GC patients (**Figure 3G**).

**Table 3:** Correlations between the expression level of tRF-Glu-TTC-027 and the clinicopathological features in 33 pairs of GC tissues.

Characteristics		Case	tRF-Glu-TTC-027 expression		p-value
			Low	High
All		33	21	12	
Age (years)	< 65	21	13	8	0.544
	>= 65	12	8	4
Gender	Male	26	18	8	0.198
	Female	7	3	4
Size (cm)	< 7.5	16	7	9	0.032*
	>= 7.5	17	14	3
Histology	Poor-differentiated	14	12	2	0.033*
	well-differentiated	19	9	10
TNM stage	I-II	7	5	2	0.494
	III-IV	26	16	10

*p < 0.05.

### tRF-Glu-TTC-027 Inhibited the Progression of GC Cells *In Vitro*

**Figure 4:**
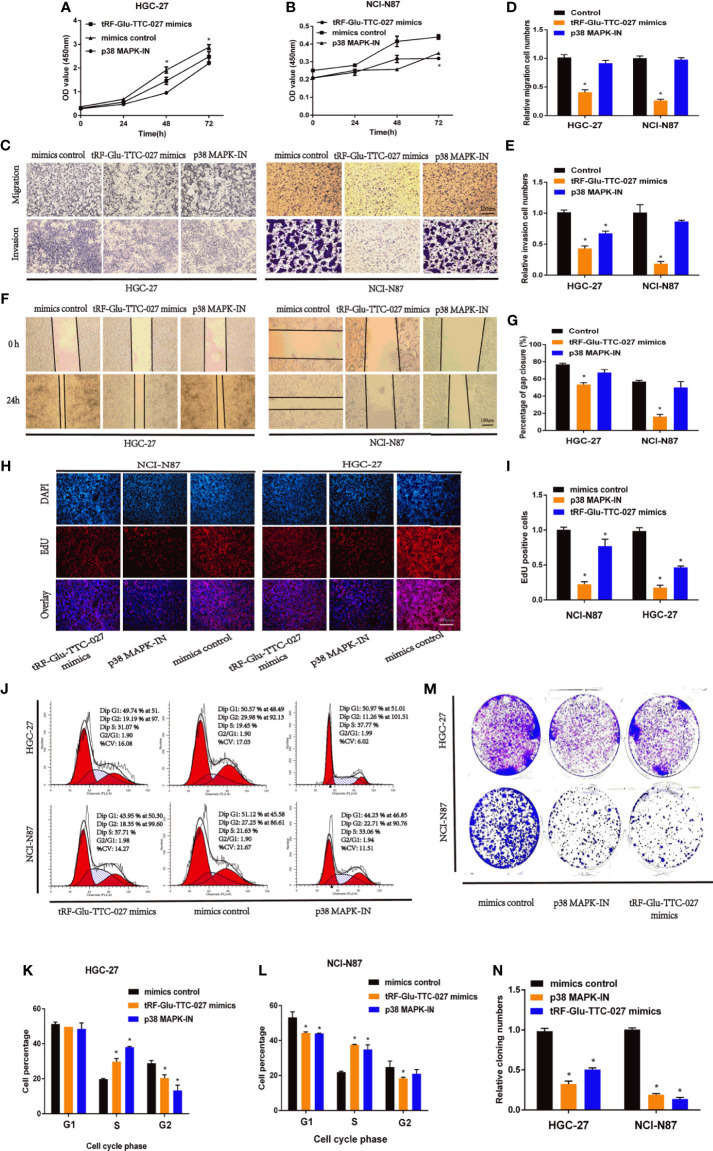
tRF-Glu-TTC-027 declined the biological function of gastric cancer *in vitro*. **(A, B)** The CCK-8 assay indicated that tRF-Glu-TTC-027 and p38 MAPK pathway inhibitor (p38 MAPK-IN) could effectively decline the proliferation of NCI-N87 and HGC-27. **(C–E)** tRF-Glu-TTC-027 was capable of suppressing the migration and invasion capacities of NCI-N87 and HGC-27 cell lines. **(F, G)** Through the observation and calculation of the cell gap closure, we demonstrated that tRF-Glu-TTC-027 was capable of fully inhibiting the migration ability of GC cells in the wound healing assays. **(H, I)** EdU assays showed that tRF-Glu-TTC-027 and p38 MAPK-IN formed fewer DNA replication levels than the control group. **(J–L)** The impact of tRF-Glu-TTC-027 and p38 MAPK-IN on GC cell cycle profile using flow cytometry. **(M, N)** The obvious change in the number of cell clones in the colony formation assays fully confirmed the inhibitory effect of tRF-Glu-TTC-027 and p38 MAPK-IN on GC. (*P < 0.05, Student’s t-test. Scale bar = 100 µm).

We transferred tRF-Glu-TTC-027 mimics, mimics control, or p38 MAPK pathway inhibitor (p38 MAPK-IN as the positive control) into the GC cells to discover the biological characteristics of tRF-Glu-TTC-027 in GC cell lines. The CCK-8 assay indicated that tRF-Glu-TTC-027 and p38 MAPK-IN could effectively decline the GC proliferation of great significance (**Figures 4A, B** **)**. In addition, tRF-Glu-TTC-027 was capable of suppressing the migration and invasion capacities of GC cell lines (**Figures 4C–E** **)**. Then the wound healing assay was applied to further demonstrate the effect of tRF-Glu-TTC-027 and p38 MAPK-IN on the GC cells, and the result was consistent with the previous assays (**Figures 4F, G** **)**. Moreover, EdU assays also showed that tRF-Glu-TTC-027 and p38 MAPK-IN formed less DNA replication level than the control group, which was reflected in the aspect of the fluorescence concentration of EdU (**Figures 4H, I** **)**. To differentiate and analyze the impact of tRF-Glu-TTC-027 on the GC cell cycle, we transfected cells with tRF-Glu-TTC-027 mimics, mimics control, or p38 MAPK-IN and tested the distribution of cell cycle with flow cytometry. Compared with the mimics control group, the ratio in the S phase of the tRF-Glu-TTC-027 mimics group significantly increased from 19.45% to 31.07%, p38 MAPK-IN group increased from 19.45% to 37.77%, but in the G2 phase of tRF-Glu-TTC-027 mimics group significantly decreased from 29.98% to 19.19%, p38 MAPK-IN group decreased from 29.98% to 11.26% in HGC-27 cells. Analogously, the cells in the G1 phase of the tRF-Glu-TTC-027 mimics group significantly declined from 51.12% to 43.95%, p38 MAPK-IN group declined from 51.12% to 44.23%, the cells in the S phase of the tRF-Glu-TTC-027 mimics group increased from 21.63% to 37.71%, p38 MAPK-IN group increased from 21.63% to 33.06%, whereas the cells in the G2 phase decreased from 27.25% to 18.35%, p38 MAPK-IN group decreased from 27.25% to 22.71% in NCI-N87 cells (**Figures 4J–L** **)**. Therefore, tRF-Glu-TTC-027 and p38 MAPK-IN can arrest the cell cycle of NCI-N87 and HGC-27 at the S phase to some extent. Then the obvious change in the number of cell clones in the colony formation assays fully confirmed the inhibitory effect of tRF-Glu-TTC-027 and p38 MAPK-IN on GC (**Figures 4M, N** **)**.

### The Rescue Assays Were Performed to Evaluate the Regulative Relationship Between tRF-Glu-TTC-027 and p38 MAPK Pathway Inhibitor (p38 MAPK-IN) in HGC-27 and NCI-N87

**Figure 5:**
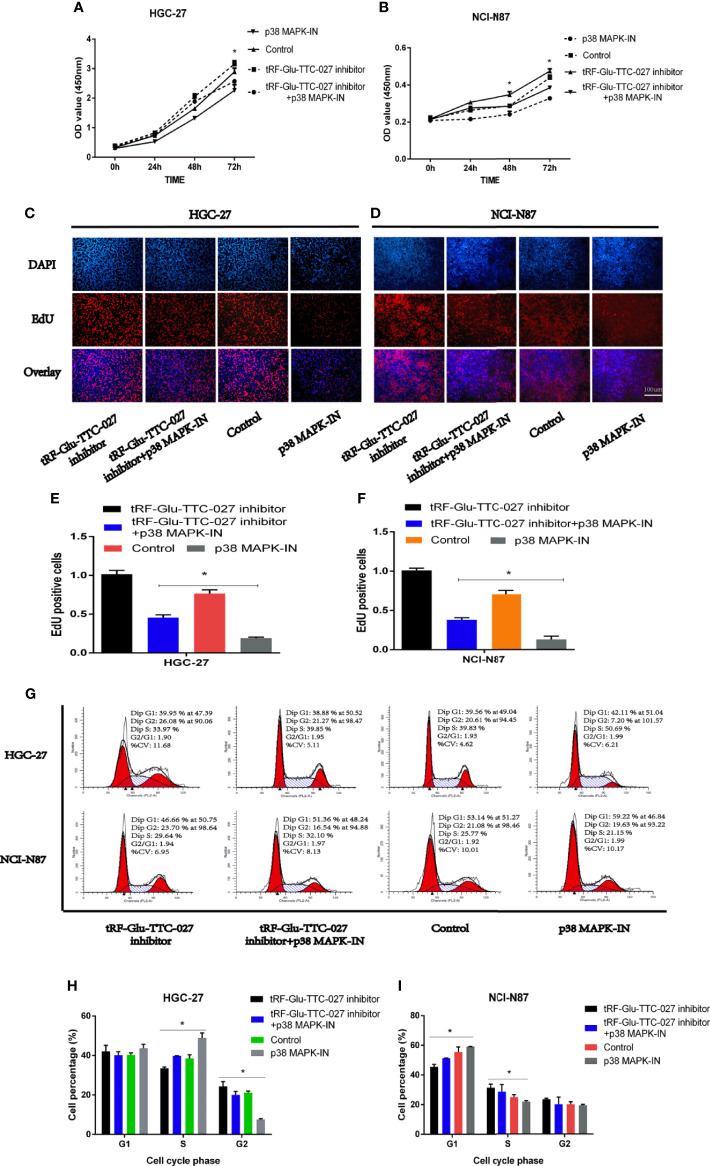
The rescue assays were performed to evaluate the regulative relationship between tRF-Glu-TTC-027 and p38 MAPK pathway inhibitor (p38 MAPK-IN) in HGC-27 and NCI-N87. **(A, B)** tRF-Glu-TTC-027 inhibitor facilitated the proliferation of HGC-27 and NCI-N87, and the inhibitory effect of p38 MAPK-IN could be reversed by tRF-Glu-TTC-027 inhibitor. **(C–F)** EdU assay demonstrated that tRF-Glu-TTC-027 inhibitor promoted the DNA replication in GC cell lines, while p38 MAPK-IN significantly suppressed the EdU assimilation and could be reversed by tRF-Glu-TTC-027 inhibitor during the proliferative process. **(G–I)** p38 MAPK-IN imposed the inhibitory effect on the GC cells to cause the cell cycle arrest and this was able to be relieved by tRF-Glu-TTC-027 inhibitor. (*P < 0.05, Student’s t-test. Scale bar = 100 µm).

To further elucidate the regulative function of tRF-Glu-TTC-027 on GC, we undertook the rescue assays with tRF-Glu-TTC-027 inhibitor group, tRF-Glu-TTC-027 inhibitor + p38 MAPK-IN group, p38 MAPK-IN group, and control group. In the CCK8 assay, tRF-Glu-TTC-027 inhibitor facilitated the proliferation of HGC-27 and NCI-N87, and this stimulative effect could be reversed by p38 MAPK-IN **(** **Figures 5A, B** **)**. We demonstrated that tRF-Glu-TTC-027 inhibitor could promote the DNA replication in GC cell lines, while p38 MAPK-IN significantly suppressed the EdU assimilation and could reverse the stimulative effect of tRF-Glu-TTC-027 inhibitor during the proliferative process in EdU assay **(** **Figures 5C–F** **)**. Then flow cytometry was used to analyze the cell cycle, and we found that p38 MAPK-IN imposed the inhibitory effect on the GC cells to cause the cell cycle arrest and this was able to be relieved by tRF-Glu-TTC-027 inhibitor **(** **Figures 5G–I** **)**.

### tRF-Glu-TTC-027 Influenced the Expression of Related Proteins in the MAPK Signaling Pathway

**Figure 6:**
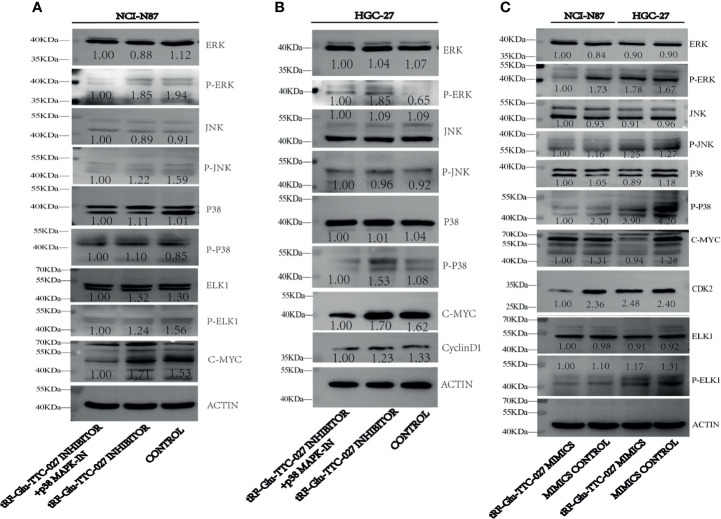
tRF-Glu-TTC-027 influenced the expression of related proteins in the MAPK signaling pathway. **(A, B)** tRF-Glu-TTC-027 inhibitor could upregulate the expression of p-p38 and c-Myc in HGC-27, and p-ERK, p-p38, and c-Myc in NCI-N87. p38 MAPK-IN could suppress the expression of p-ERK, p-p38, Elk1, p-Elk1, c-Myc, cyclinD1 in HGC-27 and NCI-N87. **(C)** tRF-Glu-TTC-027 mimics could inhibit the expression of p-ERK, p-JNK, p-p38, c-Myc, CDK2, p-ELK1 in NCI-N87, and p38, p-p38, c-Myc, p-ELK1 in HGC-27.

**Figure 7:**
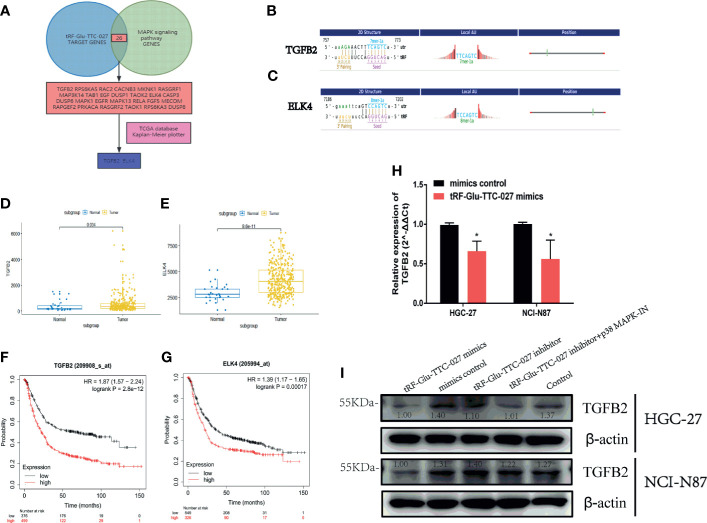
tRF-Glu-TTC-027 regulated the MAPK signaling pathway significantly. **(A)** we discovered 26 possible target genes by taking the intersection of tRF-Glu-TTC-027 target genes and genes in the MAPK signaling pathway. **(B, C)** *TGFB2* and *ELK4* were selected as their 3’ UTR owned the target sites with tRF-Glu-TTC-027. **(D, E)** *TGFB2* and *ELK4* were highly expressed in GC compared with NATs. **(F, G)** *TGFB2* and *ELK4* were associated with poor prognoses. **(H, I)** RNA and protein levels of *TGFB2* were regulated by tRF-Glu-TTC-027 to varying degrees. *P < 0.05, Student’s t-test.

To figure out the specific proteins influenced by tRF-Glu-TTC-027, we adopted immunoblotting to test the expression levels of proteins in the MAPK signaling pathway. And we found that tRF-Glu-TTC-027 inhibitor could upregulate the expression of p-p38 and c-Myc in HGC-27, and p-ERK, p-p38, and c-Myc in NCI-N87. p38 MAPK-IN could suppress the expression of p-ERK, p-p38, Elk1, p-Elk1, c-Myc, cyclinD1 in HGC-27 and NCI-N87 **(** **Figures 6A, B** **)**. tRF-Glu-TTC-027 mimics could inhibit the expression of p-ERK, p-JNK, p-p38, c-Myc, CDK2, p-ELK1 in NCI-N87, and p38, p-p38, c-Myc, p-ELK1 in HGC-27 (**Figure 6C**). To further investigate the underlying mechanism that tRF-Glu-TTC-027 influenced the MAPK signaling pathway, we discovered 26 possible target genes by taking the intersection of tRF-Glu-TTC-027 target genes and genes in the MAPK signaling pathway (**Figure 7A**). Then we analyzed the TCGA database and the Kaplan Meier plotter website to screen the significant targets. *TGFB2* and *ELK4* were selected as their 3’ UTR owned the target sites with tRF-Glu-TTC-027 (**Figures 7B, C**), and were highly expressed in GC compared with NATs (**Figures 7D, E**). Meanwhile, *TGFB2* and *ELK4* were associated with poor prognosis (**Figures 7F, G**). Finally, PCR and immunoblotting were conducted, and we found that *TGFB2* was regulated by tRF-Glu-TTC-027 in HGC-27 and NCI-N87 (**Figures 7H, I**).

### tRF-Glu-TTC-027 Decreased Tumor Growth in NCI-N87 Xenografts

**Figure 8:**
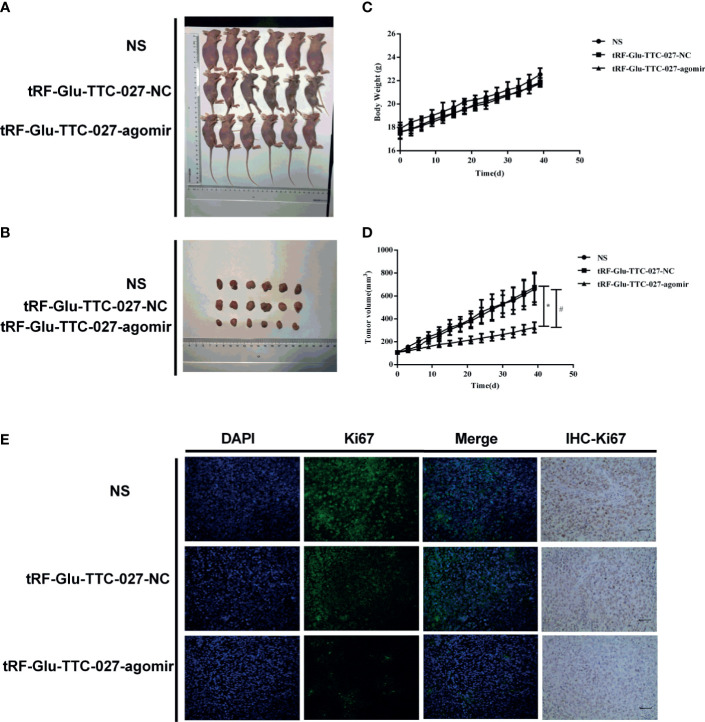
tRF-Glu-TTC-027 decreased tumor growth in NCI-N87 xenografts. **(A, B)** Representative images of NCI-N87 xenografts *in vivo*. Six mice are included in each group. **(C, D)** The data of body weight and tumor volume in each indicated group. tRF-Glu-TTC-027 significantly suppressed tumor growth. And there was no significant difference among these three groups in terms of the bodyweight of the mice. **(E)** We then conducted the immunofluorescence assay and IHC assay for Ki67 to further confirm the proliferative activity of xenografts. The fluorescence density of Ki67 decreased significantly in the tRF-Glu-TTC-027 agomir group compared with NS and tRF-Glu-TTC-027 NC group, and the result was consistent with the IHC assay. (*^#^P < 0.05, Student’s t-test. Scale bar = 100 µm).

The *in vivo* effect of tRF-Glu-TTC-027 was evaluated using NCI-N87 xenografts. The results showed that tRF-Glu-TTC-027 significantly reduced the capacity of the tumor growth (**Figures 8A, B**). However, no significant difference was observed among these three groups concerning the bodyweight of the mice (**Figure 8C**). The tumor volume in the NS group increased from 106.733 ± 2.400 mm^3^ to 659.044 ± 136.553 mm^3^, in the tRF-Glu-TTC-027 NC group from 106.929 ± 3.263 mm^3^ to 676.301 ± 130.231 mm^3^, and in the tRF-Glu-TTC-027 agomir group from 107.618 ± 8.491 mm^3^ to 324.807 ± 45.991 mm^3^ (**Figure 8D**). The volume of the explant tumors in the tRF-Glu-TTC-027 agomir group was smaller than those in the NS and tRF-Glu-TTC-027 NC group at the endpoint of the experiment significantly (**Figure 8B**). We then conducted the immunofluorescence assay and IHC assay for Ki67 to further confirm the proliferative activity of xenografts. As shown in **Figure 8E**, the fluorescence density of Ki67 decreased significantly in the tRF-Glu-TTC-027 agomir group compared with NS and tRF-Glu-TTC-027 NC group, and the result was consistent with the IHC assay.

## Discussion

tsRNAs are derived from the family of non-coding RNAs and are widely present in the biological process (8). They are involved in the cell cycle, proliferation, cell apoptosis, and many other biological regulation pathways (15). Many pieces of literature reported that tRFs are actively derived from precursor RNAs and the 3’ or 5’ end-matching tRFs are largely cleaved by Dicer1 owing to their structural features (7, 16). Meanwhile, considering the bind method between tRFs and mRNA of the target genes, Kuscu et al. showed that Dicer-independent tRF-3s could suppress the posttranscription of genes by an Argonaute-RISC method, which had a certain degree of similarity with miRNAs (17). In addition, abnormally expressed tsRNAs can act as tumor suppressor genes or oncogenes in carcinoma through multivariate mechanisms to regulate the progression of carcinomas (11, 12, 18, 19). Several studies have found that tRFs can regulate the activity of kinases to promote the proliferation of cancer cells (13, 20). Although the roles of tRFs have been demonstrated in many kinds of tumors, GC was rarely involved.

In this study, high-throughput sequencing was conducted in six pairs of GC tissues. Furthermore, 69 up-regulated and 42 down-regulated tsRNAs were discovered successfully. By expanding the amount of GC tissues to conduct the RT-PCR analysis, we selected tRF-Glu-TTC-027 as the research target. Meanwhile, bioinformatic analysis was applied to uncover the potential pathway that was able to best explain the biological characteristics of GC cell lines after the transfection of tRF-Glu-TTC-027 mimics. Interestingly, the MAPK signaling pathway successfully attracted our attention after the analysis of GO and KEGG databases. We then postulated that tRF-Glu-TTC-027 might regulate the progression of GC with the MAPK signaling pathway. Next, assays *in vitro* and *in vivo* were undertaken to demonstrate this hypothesis. We then conducted the analysis of RT-PCR results and confirmed that tRF-Glu-TTC-027 was significantly associated with histological grade and tumor size of GC. The Sanger sequencing and the gel electrophoresis were used to confirm the sequence of tRF-Glu-TTC-027. Subsequently, we used the FISH assay to demonstrate that tRF-Glu-TTC-027 was mainly distributed in the cytoplasm. Afterward, further exploration indicated that tRF-Glu-TTC-027 could significantly suppress the advancement of GC *in vitro* and *in vivo*. It should be pointed out that tRF-Glu-TTC-027 could significantly regulate the relevant proteins in the MAPK signaling pathway which exerted an influence on the oncology characteristics of GC cell lines to some extent. Hence, these findings revealed a remarkable function of tRF-Glu-TTC-027 as a tumor suppressor in GC. In summary, these results were consistent with our hypothesis that tRF-Glu-TTC-027 regulated the progression of GC with the MAPK signaling pathway.

Compared with our results, a considerable amount of studies were consistent with the present research concerning the function of tsRNAs. Recent studies discovered that tsRNAs are related to some kinds of tumors (21–24). Mo et al. uncovered that 5-tiRNA^Val^ was able to act as an inhibitor in breast cancer (12). Analogously, a novel class of tRFs influenced the YBX1 UTRs by regulating the stability of multiple oncogenic transcripts (6). Falconi reported that tRF3E could inhibit breast cancer *via* the NCL-mediated mechanism (11). However, some studies also reported several contrary results in terms of the biological characteristics of tsRNAs. Zhang et al. unfolded that tRF-03357 might promote the progression of HGSOC by regulating *HMBOX1* (13). Huang et al. elaborated that miR-1280 was influenced by tRFs in the cancer stem-like cells *via* the Notch signaling pathway in colorectal cancer (19). Our present study screened the MAPK signaling pathway by bioinformatic analysis, and this was further confirmed by immunoblotting. Analogously, some other pathways, related to tsRNAs, had also been reported by a few studies (25, 26). With regard to the MAPK signaling pathway, it has been uncovered and widely accepted as a pathway that could promote the progression of tumors (27–31). And during the process of this research, we verified that all the three classical pathways of the MAPK pathway including ERK1/2, JNK, p38 were regulated by tRF-Glu-TTC-027 to varying degrees. Interestingly, we did not find a significant correlation between tRF-Glu-TTC-027 and the prognosis of GC patients in this study. It might result from the deficiency of the GC specimens.

In this study, we applied high-throughput sequencing to discover novel diagnostic and therapeutic targets for GC, and tRF-Glu-TTC-027 was preliminarily explored. However, the further mechanism tRF-Glu-TTC-027 was involved in needs to be investigated thoroughly. For instance, the relationship between tRF-Glu-TTC-027 and *TGFB2* needs to be further explored in the subsequent research. Moreover, whether the biological characteristics were only regulated by tRF-Glu-TTC-027 or not should be figured out using multiple methods. Meanwhile, the findings in this study have not fully elucidated the effects of other signaling pathways which were demonstrated to promote the progression of GC.

In summary, our study firstly elaborated the function of tRF-Glu-TTC-027 in GC. The results indicated that tRF-Glu-TTC-027 was significantly down-regulated in GC and associated with histological grade and tumor size in the aspect of pathology analysis. Furthermore, we identified that tRF-Glu-TTC-027 could suppress the progression of GC through inhibition of the MAPK signaling pathway. Taken together, tRF-Glu-TTC-027 could be a potential target for molecular therapy in GC.

## Data Availability Statement

The original contributions presented in the study are included in the article/**Supplementary Material**. Further inquiries can be directed to the corresponding authors.

## Ethics Statement

The animal study was reviewed and approved by The Affiliated Cancer Hospital of Nanjing Medical University.

## Author Contributions

WX, FY, and HQC: conception and design. WX, BZ, HHC, and JuW: acquisition of data. WX, JiW, JZ, and QH: analysis and interpretation of data. WX and LT: writing and review of the manuscript. FY and HQC: study supervision. All authors contributed to the article and approved the submitted version.

## Funding

This research was supported by the National Key Research and Development Program (NO. 2017YFC0908300), Jiangsu Provincial Key Research and Development Program (NO. BE2018750), a grant from the General Program of Jiangsu cancer hospital (NO. ZM202003).

## Conflict of Interest

The authors declare that the research was conducted in the absence of any commercial or financial relationships that could be construed as a potential conflict of interest.

## Publisher’s Note

All claims expressed in this article are solely those of the authors and do not necessarily represent those of their affiliated organizations, or those of the publisher, the editors and the reviewers. Any product that may be evaluated in this article, or claim that may be made by its manufacturer, is not guaranteed or endorsed by the publisher.
