# Supplementary material for: tRNA-Derived Fragment tRF-Glu-TTC-027 Regulates the Progression of Gastric Carcinoma via MAPK Signaling Pathway
Source: Front Oncol. 2021 Aug 23;11:733763. doi: 10.3389/fonc.2021.733763 (PMC8419445; doi:10.3389/fonc.2021.733763)
Supplement: Supplementary file 1 [file DataSheet_1.docx]

**Supplementary Table 1** Significantly up-regulated tRNA-derived fragments based on high-throughput sequencing.

| **tRF_ID** | **tRF_Seq** | **Type** | **tRFdb_ID** | **MINTbase_ID** | **Length** |
| --- | --- | --- | --- | --- | --- |
| tRF-Ser-AGA-017 | GTAGTCGTGGCCGAGTGGTTAAGGTGATGGAC | tRF-5c | - | - | 32 |
| tiRNA-His-GTG-001 | GCCGTGATCGTATAGTGGTTAGTACTCTGCGTTG | tiRNA-5 | - | tRF-34-PW5SVP9N15WV2P | 34 |
| tiRNA-Val-TAC-003 | GGTTCCATAGTGTAGTGGTTATCACATCTGCTTT | tiRNA-5 | - | - | 34 |
| tRF-Glu-TTC-017 | TCCCATATGGTCTAGCGGTTAGGATTCC | tRF-5c | - | tRF-28-86V8WPMN1E0J | 28 |
| tRF-Val-TAC-094 | TGGTGTGGTCTGTTGTTTT | tRF-1 | - | - | 19 |
| tiRNA-Gly-GCC-002 | GCATGGGTGGTTCAGTGGTAGAATTCTCGCCTG | tiRNA-5 | - | tRF-33-P4R8YP9LON4VDP | 33 |
| tRF-Gly-TCC-005 | GCGTTGGTGGTATAGTGGTAAGCATAGCT | tRF-5c | - | - | 29 |
| tRF-Glu-TTC-018 | TCCCATATGGTCTAGCGGTTAGGATTCCT | tRF-5c | - | tRF-29-86V8WPMN1EJ3 | 29 |
| tRF-Gly-GCC-036 | TCCCGGCCCATGCACCA | tRF-3a | 3028a | tRF-17-8SP6X52 | 17 |
| tiRNA-Val-CAC-001 | GCTTCTGTAGTGTAGTGGTTATCACGTTCGCCTC | tiRNA-5 | - | tRF-34-Q99P9P9NH57S15 | 34 |
| tRF-Gln-CTG-014 | GGTTCCATGGTGTA | tRF-5a | 5021a | - | 14 |
| tRF-Glu-TTC-069 | TCCCTGGTGGTCTAGTGGCTAGGATTCGG | tRF-5c | - | tRF-29-87R8WP9I1EJ4 | 29 |
| tRF-Glu-TTC-009 | TCCCACATGGTCTAGCGGTTAGGATTCC | tRF-5c | - | tRF-28-86J8WPMN1E0J | 28 |
| tiRNA-Asp-GTC-001 | TCCTCGTTAGTATAGTGGTGAGTATCCCCGCCTG | tiRNA-5 | - | tRF-34-897PVP941QKS1P | 34 |
| tiRNA-Pro-AGG-001 | GGCTCGTTGGTCTAGGGGTATGATTCTCGCTTA | tiRNA-5 | - | - | 33 |
| tRF-His-GTG-005 | GCCGTGATCGTATAGTGGTTAGTACTCT | tRF-5c | - | tRF-28-PW5SVP9N1503 | 28 |
| tRF-Gly-TCC-009 | GCGTTGGTGGTATAGTGGTGAGCATAGCT | tRF-5c | - | tRF-29-QNR8VP94FQEW | 29 |
| tiRNA-Lys-CTT-002 | GCCCGGCTAGCTCAGTCGGTAGAGCATGAGACTC | tiRNA-5 | - | tRF-34-PSQP4PW3FJI0E5 | 34 |
| tRF-Glu-TTC-010 | TCCCACATGGTCTAGCGGTTAGGATTCCT | tRF-5c | - | tRF-29-86J8WPMN1EJ3 | 29 |
| tRF-Gly-TCC-008 | GCGTTGGTGGTATAGTGGTGAGCATAGC | tRF-5c | - | tRF-28-QNR8VP94FQ9 | 28 |
| tRF-Gly-GCC-008 | GCATGGGTGGTTCAGTGGTAGAATTCTCG | tRF-5c | - | tRF-29-P4R8YP9LONHK | 29 |
| tRF-Gly-TCC-014 | GCGTTGGTGGTATAGTGGTTAGCATAGCTG | tRF-5c | - | tRF-30-QNR8VP9NFQFY | 30 |
| tRF-Glu-CTC-006 | TCCCTGGTGGTCTAGTGGTTAGGATTCG | tRF-5c | - | tRF-28-87R8WP9N1E0K | 28 |
| tRF-Gly-TCC-013 | GCGTTGGTGGTATAGTGGTTAGCATAGCT | tRF-5c | - | tRF-29-QNR8VP9NFQEW | 29 |
| tiRNA-Glu-TTC-002 | TCCCATATGGTCTAGCGGTTAGGATTCCTGGTTT | tiRNA-5 | - | tRF-34-86V8WPMN1E8Y2Q | 34 |
| tRF-Gly-GCC-011 | GCATGGGTGGTTCAGTGGTAGAATTCTCGCCT | tRF-5c | - | tRF-32-P4R8YP9LON4V3 | 32 |
| tRF-Gly-TCC-012 | GCGTTGGTGGTATAGTGGTTAGCATAGC | tRF-5c | - | tRF-28-QNR8VP9NFQ9 | 28 |
| tRF-Glu-TTC-068 | TCCCTGGTGGTCTAGTGGCTAGGATTCG | tRF-5c | - | tRF-28-87R8WP9I1E0K | 28 |
| tRF-Gly-GCC-009 | GCATGGGTGGTTCAGTGGTAGAATTCTCGC | tRF-5c | - | tRF-30-P4R8YP9LON4V | 30 |
| tRF-Val-CAC-007 | GCTTCTGTAGTGTAGTGGTTATCACGTT | tRF-5c | - | tRF-28-Q99P9P9NH50E | 28 |
| tRF-Gly-GCC-010 | GCATGGGTGGTTCAGTGGTAGAATTCTCGCC | tRF-5c | 5003c | tRF-31-P4R8YP9LON4VD | 31 |
| tRF-Gly-GCC-007 | GCATGGGTGGTTCAGTGGTAGAATTCTC | tRF-5c | - | tRF-28-P4R8YP9LOND5 | 28 |
| tRF-Glu-TTC-019 | TCCCATATGGTCTAGCGGTTAGGATTCCTG | tRF-5c | - | tRF-30-86V8WPMN1E8Y | 30 |
| tiRNA-Lys-CTT-005 | GCCCAGCTAGCTCAGTCGGTAGAGCATGAGACTC | tiRNA-5 | - | - | 34 |
| tRF-Phe-GAA-021 | GTTTATGTAGCTTACC | tRF-5a | - | tRF-16-7X9PN5D | 16 |
| tRF-Lys-TTT-009 | GCCCGGATAGCTCAGTCGGTAGAGCATCAG | tRF-5c | - | tRF-30-PS5P4PW3FJHP | 30 |
| tRF-His-GTG-006 | GCCGTGATCGTATAGTGGTTAGTACTCTG | tRF-5c | - | tRF-29-PW5SVP9N15JP | 29 |
| tRF-His-GTG-007 | GCCGTGATCGTATAGTGGTTAGTACTCTGC | tRF-5c | - | tRF-30-PW5SVP9N15WV | 30 |
| tRF-Asp-GTC-034 | TCCTCGTTAGTATAGTGGTGAGTATCCC | tRF-5c | - | tRF-28-897PVP941QDJ | 28 |
| tRF-Lys-CTT-005 | GCCCGGCTAGCTCAGTCGGTAGAGCATGA | tRF-5c | - | tRF-29-PSQP4PW3FJFL | 29 |
| tRF-Gly-TCC-046 | GCGTTGGTGGTATAGTGGTGAGCATAGT | tRF-5c | - | - | 28 |
| tRF-Gly-TCC-044 | GCGTTGGTGGTATAGTGGTGAGCATAGCTG | tRF-5c | 5008c | tRF-30-QNR8VP94FQFY | 30 |
| tiRNA-Pro-CGG-001 | GGCTCGTTGGTCTAGGGGTATGATTCTCGCTTC | tiRNA-5 | - | tRF-33-6978WPRLXN4V05 | 33 |
| tRF-Lys-CTT-007 | GCCCGGCTAGCTCAGTCGGTAGAGCATGAGA | tRF-5c | - | tRF-31-PSQP4PW3FJI0B | 31 |
| tiRNA-Val-TAC-002 | GGTTCCATAGTGTAGCGGTTATCACGTCTGCTTT | tiRNA-5 | - | - | 34 |
| tRF-Val-CAC-008 | GCTTCTGTAGTGTAGTGGTTATCACGTTC | tRF-5c | - | tRF-29-Q99P9P9NH525 | 29 |
| tRF-Lys-TTT-008 | GCCCGGATAGCTCAGTCGGTAGAGCATCA | tRF-5c | - | tRF-29-PS5P4PW3FJF2 | 29 |
| tRF-Lys-CTT-006 | GCCCGGCTAGCTCAGTCGGTAGAGCATGAG | tRF-5c | - | tRF-30-PSQP4PW3FJI0 | 30 |
| tRF-Glu-CTC-007 | TCCCTGGTGGTCTAGTGGTTAGGATTCGG | tRF-5c | - | tRF-29-87R8WP9N1EJ4 | 29 |
| tiRNA-Lys-CTT-001 | GCCCGGCTAGCTCAGTCGGTAGAGCATGAGACCC | tiRNA-5 | - | tRF-34-PSQP4PW3FJI0EJ | 34 |
| tRF-Glu-TTC-024 | TCCCTGGTGGTCTAGTGGCTAGGATTCGGCG | tRF-5c | - | - | 31 |
| tRF-Glu-TTC-020 | TCCCATATGGTCTAGCGGTTAGGATTCCTGG | tRF-5c | - | tRF-31-86V8WPMN1E8Y0 | 31 |
| tiRNA-Val-TAC-004 | GGTTCCATAGTGTAGTGGTTATCACGTCTGCTTT | tiRNA-5 | - | tRF-34-R9JP9P9NH5SYHQ | 34 |
| tiRNA-Glu-CTC-001 | TCCCTGGTGGTCTAGTGGTTAGGATTCGGCGCTC | tiRNA-5 | - | tRF-34-87R8WP9N1EWJI5 | 34 |
| tRF-His-GTG-003 | GCCGTGATCGTATA | tRF-5a | - | - | 14 |
| tRF-Glu-TTC-023 | TCCCTGGTGGTCTAGTGGCTAGGATTCGGC | tRF-5c | - | tRF-30-87R8WP9I1EWJ | 30 |
| tiRNA-Glu-TTC-003 | TCCCTGGTGGTCTAGTGGCTAGGATTCGGCGCTT | tiRNA-5 | - | tRF-34-87R8WP9I1EWJIQ | 34 |
| tiRNA-Gly-CCC-002 | GCATTGGTGGTTCAGTGGTAGAATTCTCGCCTC | tiRNA-5 | - | tRF-33-PNR8YP9LON4VD5 | 33 |
| tiRNA-Glu-TTC-001 | TCCCACATGGTCTAGCGGTTAGGATTCCTGGTTT | tiRNA-5 | - | tRF-34-86J8WPMN1E8Y2Q | 34 |
| tRF-Arg-ACG-017 | TTCGACTTTAGTGGAAACTTT | tRF-1 | - | - | 21 |
| tRF-His-GTG-008 | GCCGTGATCGTATAGTGGTTAGTACTCTGCG | tRF-5c | - | tRF-31-PW5SVP9N15WV0 | 31 |
| tRF-Lys-CTT-037 | GCCCGGCTAGCTCAGTCGGTAGAGCATGG | tRF-5c | - | tRF-29-PSQP4PW3FJF4 | 29 |
| tRF-Glu-TTC-011 | TCCCACATGGTCTAGCGGTTAGGATTCCTG | tRF-5c | - | tRF-30-86J8WPMN1E8Y | 30 |
| tRF-His-GTG-009 | GCCGTGATCGTATAGTGGTTAGTACTCTGCGT | tRF-5c | - | tRF-32-PW5SVP9N15WVN | 32 |
| tiRNA-Lys-TTT-002 | GCCCGGATAGCTCAGTCGGTAGAGCATCAGACTT | tiRNA-5 | - | tRF-34-PS5P4PW3FJHPEQ | 34 |
| tRF-His-GTG-028 | GCCATGATCGTATAGTGGTTAGTACTCTGCGC | tRF-5c | - | - | 32 |
| tRF-Lys-CTT-008 | GCCCGGCTAGCTCAGTCGGTAGAGCATGAGAC | tRF-5c | - | tRF-32-PSQP4PW3FJI01 | 32 |
| tiRNA-Gly-GCC-003 | GCATTGGTGGTTCAGTGGTAGAATTCTCGCCTG | tiRNA-5 | - | tRF-33-PNR8YP9LON4VDP | 33 |
| tRF-Lys-CTT-004 | GCCCGGCTAGCTCAGTCGGTAGAGCATG | tRF-5c | - | tRF-28-PSQP4PW3FJD0 | 28 |

**Supplementary Table 2** Significantly down-regulated tRNA-derived fragments based on high-throughput sequencing.

| **tRF_ID** | **tRF_Seq** | **Type** | **tRFdb_ID** | **MINTbase_ID** | **Length** |
| --- | --- | --- | --- | --- | --- |
| tRF-Val-CAC-016 | AAGTGGTTCCCGTTT | tRF-1 | - | - | 15 |
| tRF-Val-CAC-023 | AAGTGGTTCCTGTT | tRF-1 | - | - | 14 |
| tRF-Val-CAC-017 | AAGTGGTTCCCGTTTT | tRF-1 | - | - | 16 |
| tRF-Pro-AGG-011 | AATCCCGGACGAGCCCCCA | tRF-3b | - | tRF-19-DRMD5112 | 19 |
| tRF-Ser-GCT-114 | GAGAAAGCTCACAAGAACTGCTAACTCATGCC | tRF-5c | - | tRF-32-5BF900BY4D84J | 32 |
| tRF-Glu-TTC-073 | TGACTGGACCTTTCTTTT | tRF-1 | - | - | 18 |
| tiRNA-Phe-GAA-001 | GTTTATGTAGCTTACCTCCTCAAAGCAATACACTG | tiRNA-5 | - | - | 35 |
| tRF-Lys-TTT-139 | CACTGTAAAGCTAACTTAGCATTAACCTTTTA | tRF-5c | - | - | 32 |
| tRF-Ala-AGC-057 | TCAATCCCCGGCACCTCCACCA | tRF-3b | - | tRF-22-8EKSP1852 | 22 |
| tRF-Glu-TTC-027 | TGACTGGACCTTTCTTT | tRF-1 | - | - | 17 |
| tRF-Gly-CCC-002 | GCATTGGTGGTTCA | tRF-5a | - | - | 14 |
| tRF-Glu-TTC-026 | TCGACTCCCGGTGTGGGAACCA | tRF-3b | 3015b | tRF-22-WD8S746D2 | 22 |
| tRF-Gly-CCC-035 | GCATTGGTGGTTCAG | tRF-5a | - | - | 15 |
| tRF-Asn-GTT-016 | GTCAGTGCTTCTTT | tRF-1 | - | - | 14 |
| tRF-Gly-CCC-031 | GCATTGGTGGTTCAA | tRF-5a | - | - | 15 |
| tRF-Ser-TGA-022 | GAAAAAGTCATGGAGG | tRF-5a | - | tRF-16-OB16900 | 16 |
| tRF-Ala-AGC-008 | GGGGATGTAGCTCAGTGGTAGAGCGCATGCTT | tRF-5c | - | tRF-32-R29P4P9L5HJVQ | 32 |
| tRF-Ser-TGA-011 | GCAGCGATGGCCGAGT | tRF-5a | - | - | 16 |
| tRF-Ser-GCT-006 | GACGAGGTGGCCGAGT | tRF-5a | - | tRF-16-OUR830E | 16 |
| tRF-Cys-GCA-283 | AGCTCCGAGGTGATTTTCATATTGAATTGC | tRF-5c | - | tRF-30-F9LKXNYQIUIV | 30 |
| tRF-Asn-GTT-017 | GTCAGTGCTTCTTTT | tRF-1 | - | - | 15 |
| tiRNA-Pro-TGG-001 | CAGAGAATAGTTTAAATTAGAATCTTAGCTTT | tiRNA-5 | - | - | 32 |
| tRF-Ser-GCT-113 | GAGAAAGCTCACAAGAACTGCTAACTCATGC | tRF-5c | - | - | 31 |
| tRF-Gly-GCC-012 | TCGATTCCCGGCCAATGCACCA | tRF-3b | 3027b | tRF-22-WE8SPOX52 | 22 |
| tRF-Cys-GCA-017 | AGCTCCGAGGTGATTTTCATATTGAATT | tRF-5c | - | - | 28 |
| tRF-Val-TAC-037 | CTTGACCGCTCTGACCA | tRF-3a | - | tRF-17-NU3IND2 | 17 |
| tRF-Ala-AGC-004 | GGGGATGTAGCTCAGT | tRF-5a | - | tRF-16-R29P4PE | 16 |
| tRF-Leu-TAG-014 | ACTTTTAAAGGATAACAGCTATCCATTGGTCT | tRF-5c | - | tRF-32-EZU05OJIH6Z33 | 32 |
| tRF-Gly-CCC-006 | GCATTGGTGGTTCAGT | tRF-5a | - | tRF-16-PNR8YPE | 16 |
| tRF-Leu-AAG-004 | GGTAGCGTGGCCGAGT | tRF-5a | - | tRF-16-RPM830E | 16 |
| tRF-Ser-GCT-112 | GAGAAAGCTCACAAGAACTGCTAACTCATG | tRF-5c | - | tRF-30-5BF900BY4D84 | 30 |
| tRF-Ser-CGA-001 | GTCACGGTGGCCGAGT | tRF-5a | - | - | 16 |
| tRF-Pro-TGG-005 | CAGAGAATAGTTTAAATTAGAATCTTAGC | tRF-5c | - | - | 29 |
| tRF-Lys-TTT-138 | CACTGTAAAGCTAACTTAGCATTAACCTTTT | tRF-5c | - | - | 31 |
| tRF-Ser-TGA-025 | GAAGCGGGTGCTCTT | tRF-1 | - | - | 15 |
| tRF-Ser-TGA-007 | GAAGCGGGTGCTCTTATTT | tRF-1 | - | - | 19 |
| tRF-Gly-CCC-016 | GCGCCGCTGGTGTAGT | tRF-5a | - | tRF-16-Q1Q89PE | 16 |
| tRF-Pro-TGG-029 | TCGTGGCTACTGTTT | tRF-1 | - | - | 15 |
| tRF-Gly-CCC-015 | GCGCCGCTGGTGTA | tRF-5a | - | - | 14 |
| tRF-Leu-AAG-003 | GGTAGCGTGGCCGAGC | tRF-5a | 5019a | tRF-16-RPM830D | 16 |
| tRF-Tyr-GTA-010 | ATCCGGCTCGAAGGACCA | tRF-3a | - | tRF-18-HSQSD2D2 | 18 |
| tRF-Gln-TTG-005 | TAGGATGGGGTGTGAT | tRF-5a | - | tRF-16-V29K9UE | 16 |
